# Sperm Motility Is Modulated by F_4_-Neuroprostane via the Involvement of Ryanodine Receptors

**DOI:** 10.3390/ijms26157231

**Published:** 2025-07-26

**Authors:** Cinzia Signorini, Elena Moretti, Laura Liguori, Caterina Marcucci, Thierry Durand, Jean-Marie Galano, Camille Oger, Giulia Collodel

**Affiliations:** 1 Department of Molecular and Developmental Medicine, University of Siena, Policlinico Le Scotte, 53100 Siena, Italy; cinzia.signorini@unisi.it (C.S.); laura.liguori@student.unisi.it (L.L.); caterin.marcucci@student.unisi.it (C.M.); giulia.collodel@unisi.it (G.C.); 2 Institut des Biomolécules Max Mousseron (IBMM), Pole Chimie Balard Recherche, UMR 5247, CNRS, Université de Montpellier, ENSCM, 34090 Montpellier, France; thierry.durand@umontpellier.fr (T.D.); marie.galano@umontpellier.fr (J.-M.G.); camille.oger@umontpellier.fr (C.O.)

**Keywords:** acrosomal status, dantrolene, F_4_-neuroprostanes, ryanodine receptors, sperm motility

## Abstract

F_4_-Neuroprostanes (F_4_-NeuroPs), oxidative metabolites of docosahexaenoic acid, act as bioactive lipid mediators enhancing sperm motility and induce capacitation-like changes in vitro. Their biological action is proposed to involve sperm ion channels, in particular ryanodine receptors (RyRs), which regulate intracellular calcium homeostasis. We evaluated the effects of dantrolene, a RyR inhibitor, on motility and vitality of a selected spermatozoa at different concentrations (10, 30, 50, 100 μM). Then sperm motility, acrosome integrity, and RyR localization following co-incubation with dantrolene (D50 or D100 μM) and 4-/10-F_4t_-NeuroPs (7 ng) were investigated. Acrosomal status was assessed using *Pisum sativum* agglutinin (PSA) staining and RyR localization by immunofluorescence. D50 was identified as the minimum effective dose to induce significant reductions in sperm motility. F_4_-NeuroPs significantly increased rapid progressive motility versus controls. Co-incubation with F_4_-NeuroPs + D50 reduced rapid motility and increased in situ and circular movement. The acrosome staining appeared altered or absent to different percentages, and RyR localization was also seen in the midpiece. These findings suggested that F_4_-NeuroPs enhance sperm motility via RyR-mediated pathways, as confirmed by dantrolene inhibition. Accordingly, our results underscore the physiological relevance of RyRs in sperm function and suggest new insights into lipid-based mechanisms regulating sperm motility.

## 1. Introduction

The sperm fatty acid profile and fatty acid-derived metabolites are related to both male infertility and human sperm parameters and are proposed as potential biomarkers of infertility [1,2,3]. In addition, it is known that the sperm membrane is rich in polyunsaturated fatty acids (PUFAs), which can undergo oxidation via free radical-initiated mechanisms, leading to the release of bioactive lipid products [1]. Among these, isoprostanoids are oxygenated metabolites, isomeric to prostanoids [4].

Docosahexaenoic acid (DHA) is particularly abundant in human spermatozoa where its content is positively related to sperm motility, morphology, and concentration [5]. Following free radical-mediated oxidative metabolism, DHA is converted to F_4_-neuroprostanes (F_4_-NeuroPs), a class of isoprostanoids included in the newly named NEO-PUFAs [6].

Actually, the biological actions mediated by the products of fatty acid metabolism, including the regulation of protein ion channels and calcium influx, represent a relevant issue in the study of spermatozoa function and male infertility [3,7,8]. Interestingly, seminal F_4_-NeuroP levels were shown to be relevant to male infertility and human sperm function [9,10].

Signorini and colleagues [10] demonstrated that 7 ng of 4- and 10-F_4t_-NeuroPs (collectively defined as F_4_-NeuroPs) triggers hyperactivated sperm motility and the acrosomal reaction in rabbit and swim-up-selected human sperm. Then, it was reported that the observed effects of F_4t_-NeuroPs in selected human sperm could involve the activation of sperm ion channels, in particular the ryanodine receptor (RyR) [11].

Both molecules, 4- and 10-F_4t_-NeuroPs, belonging to the family of possible regioisomeric NeuroPs that can be formed by DHA [6], increase the progressive sperm motility, the percentage of capacitated spermatozoa [10], and the mitochondrial membrane potential [11]. Several physiological events, such as sperm hyperactivation, capacitation, and acrosome reaction, are triggered by the activation of sperm ion channels in response to different molecules. In particular, the cation channel of spermatozoa (CatSper) and potassium channels have been reported to be involved in fertilization [12]. However, RyRs are present in germ cells, and calcium mobilization through RyR channels could participate in the regulation of male germ physiology [13].

In human sperm, fertilization rate and motility are related to the RyRs [14], and the early stage of the acrosome reaction was shown to be associated with RyRs [15]. Moreover, in mice CRISP2 (cysteine-rich secretory protein 2), a sperm acrosome and tail protein can regulate calcium flow through RyRs [16].

Since 7 ng of F_4_-NeuroPs triggers sperm capacitation-like effects, demonstrated in rabbit and human spermatozoa [10] and modifies the localization of RyRs in human spermatozoa [11], in this study, we used an inhibition experiment with dantrolene to reversely verify the involvement of RyRs in biological effects on sperm motility induced by F_4_-NeuroPs.

To this aim, the in vitro effects of incubation with dantrolene, the chemical inhibitor of RyRs, and with F_4_-NeuroPs were assessed on a selected sperm population. The study included the evaluation of sperm motility and vitality, acrosomal integrity, and RyR immunolocalization.

## 2. Results

The experiments of this research were performed with 36 semen samples with normal parameters: 18 semen samples used in the first step (to investigate the effect of different dantrolene concentrations on selected sperm population) and 18 in the second one (to investigate the effect of combined treatment with dantrolene and F_4_-NeuroPs on selected sperm population). Sperm concentration, motility, morphology, and vitality of each sample resulted higher than 25th percentile (WHO, 2021) [17]. The swim-up-selected spermatozoa were incubated with dantrolene and/or F_4_-NeuroPs, and their effects on sperm motility and vitality were evaluated. Then, the acrosome status and the localization of RyRs were also assessed.

### 2.1. Effect of Dantrolene on Selected Human Sperm

**Figure 1:**
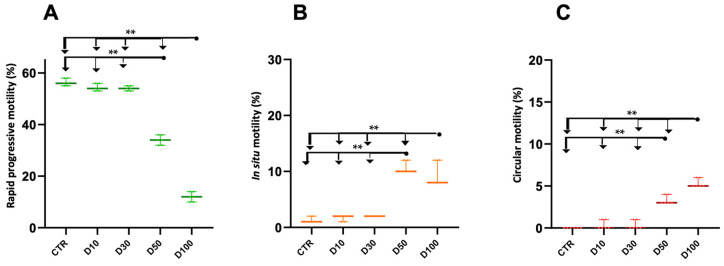
Percentage of sperm motility evaluated in upper fraction of swim-up: control sample (CTR) and in sample incubated with 10 μM, 30 μM, 50 μM, or 100 μM dantrolene (respectively D10, D30, D50, D100) for 30 min. Panels A, B, and C refer to the following: rapid progressive sperm motility (**A**), in situ sperm motility (**B**), and circular sperm motility (**C**). In each panel, differences between groups were compared using the non-parametric Kruskal–Wallis test (*p* ≤ 0.0001, *n* = 18) followed by the non-parametric Mann–Whitney test. For the Mann–Whitney test, significant *p* values are displayed in each panel. Legend: dots represent individual data; horizontal lines represent medians; and error bars represent the 95% interquartile range. ** *p* < 0.005. CTR, control; D10, D30, D50, and D100, dantrolene 10 μM, 30 μM, 50 μM, and 100 μM, respectively.

We tested the effect of different concentrations (10 μM, 30 μM, 50 μM, and 100 μM) of dantrolene (D10, D30, D50, and D100, respectively) on sperm motility and vitality of swim-up-selected human sperm (Figure 1).

The concentration of D50 was found to be the minimum effective dose and significantly reduced rapid progressive sperm motility compared with control samples and samples incubated with D10 and D30 (*p* < 0.005, Figure 1, Panel A). The opposite trend was observed for in situ sperm motility, which significantly increased in samples treated with D50 and D100 with respect to samples treated with D10 and D30 and control (*p* < 0.005, Figure 1, Panel B). In the considered samples, the presence of sperm with circular motility was quantified. Circular motility was absent in controls and in samples incubated with D10 and D30 (Figure 1, Panel C). In samples treated with D50 and D100, circular motility was significantly increased with respect to the other samples (*p* < 0.005, Figure 1, Panel C). In addition, in the samples incubated with D100, circular motility was significantly increased with respect to that observed in samples treated with D50 (*p* < 0.005, Figure 1, Panel C). No reduction in sperm vitality was observed in the analyzed samples.

### 2.2. Effect of Dantrolene on Selected Human Sperm in Presence of F_4_-NeuroPs

Swim-up-selected human sperm were divided into four aliquots: CTR, F_4_, F_4_ + D50, F_4_ + D100, as described in Materials and Methods, Step 2, and data related to sperm motility obtained with the two different concentrations of dantrolene are shown in Table 1.

Statistical analysis showed that the rapid progressive motility significantly increased after F_4_ incubation compared with that in control (*p* < 0.005) but decreased after incubation with F_4_ and dantrolene at both concentrations (D50 and D100; *p* < 0.005, Table 1); in situ motility and circular motility significantly increased at both D50 and D100 concentrations compared with those for F_4_ and CTR (*p* < 0.005, Table 1).

Thus, F_4_-NeuroPs significantly increased rapid progressive motility compared with control samples, and this effect of F_4_-NeuroPs on rapid progressive motility was turned upside down by the presence of dantrolene that exerts a dose-dependent effect on rapid progressive motility: 38% in D50 group and 8% in D100 group (Table 1).

**Figure 2:**
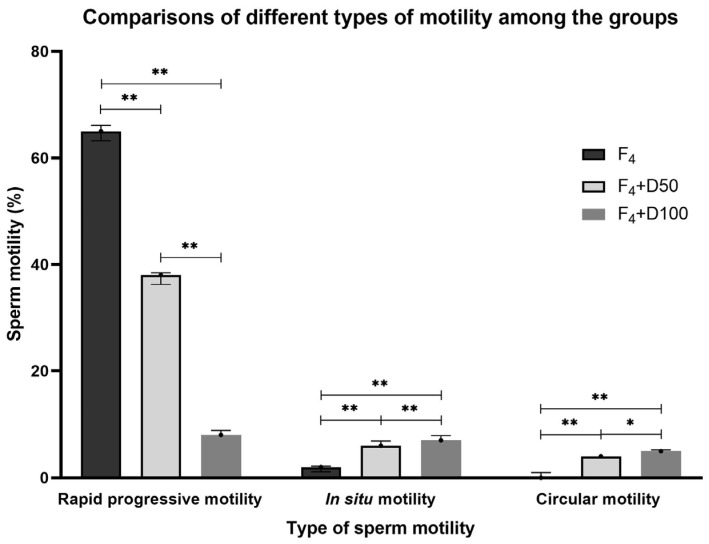
Comparison of the percentage of rapid progressive, in situ, and circular sperm motility among F_4_, F_4_ + D50, F_4_ + D100. Kruskal–Wallis test *p* < 0.0001, *n* = 18. For post hoc pairwise comparisons, the Mann–Whitney test was carried out. Data represent *p* value only for comparison between F_4_ + D50 and F_4_ + D100. The other significant comparisons are reported in Table 1. * *p* < 0.05, ** *p* < 0.005.

Finally, the counter-effects of D50 or D100 on sperm motility changes induced by F_4_-NeuroPs were compared. In Figure 2 only the significant differences between D50 and D100 are shown. D100 incubation significantly decreased progressive rapid motility (*p* < 0.005); on the contrary, in situ motility (*p* < 0.005) and circular motility (*p* < 0.05) significantly increased compared with those for D50 (Figure 2).

### 2.3. Pisum sativum Agglutinin (PSA) Evaluation and Ryanodine Receptor (RYR) Localization

Acrosome integrity and RyR localization were evaluated in CTR, F_4_, and F_4_ + D50.

**Figure 3:**
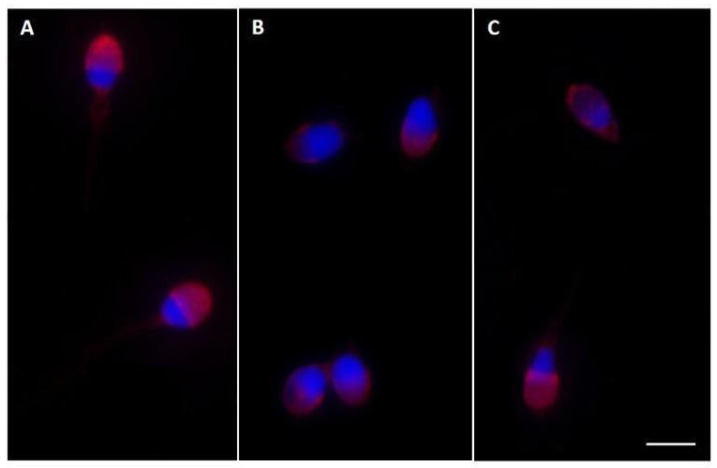
Evaluation of the acrosome status with TRITC—*Pisum sativum* agglutinin (TRITC–PSA). In (**A**), a PSA strong signal indicates intact acrosomes. The image is obtained from CTR in which the percentage of intact acrosome was 93.3%. In (**B**,**C**), the PSA label of acrosome appeared faint and sometimes absent or altered. The images were obtained after incubation with F_4_ (**B**) and with F_4_ + D50 (**C**), where the percentages of altered acrosome were increased compared with that in CTR. Nuclei (blue) were stained with DAPI. Bars: 6 μm.

In CTR, almost the totality of sperm (93.3 ± 1.53%) highlighted the PSA label in the acrosome, which appeared intact, and in the apical portion of the sperm head (Figure 3A). After incubation with F_4_ or F_4_ + D50, the percentage of intact acrosomes appeared significantly reduced (respectively, 70.6 ± 2.3% and 84.66 ± 1.15%). In sperm from F_4_ (Figure 3B) or F_4_ + D50 (Figure 3C) samples, the PSA label appeared absent (respectively, 8.3 ± 1.15% and 5 ± 1% compared with CTR, 3.6 ± 0.58%) or altered (respectively, 21 ± 1% and 10.3 ± 1.15% compared with CTR, 3 ± 1%).

**Figure 4:**
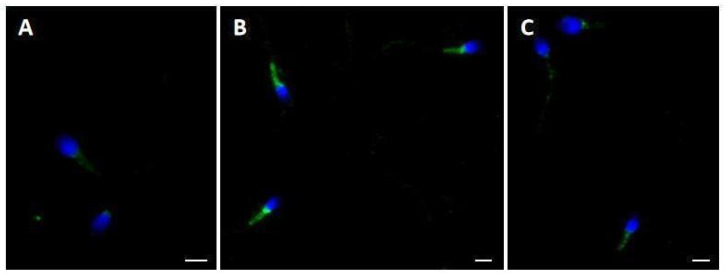
UV micrographs of swim-up-selected spermatozoa treated with anti-ryanodine antibody. In CTR, spermatozoa showed a faint labeling in the sperm tail (**A**). Spermatozoa incubated with F_4_ showed a clear localization in the midpiece (**B**). In F_4_ + D50, the labeling appeared weak (**C**). Nuclei (blue) were stained with DAPI. Bars: 6 μm.

Immunofluorescence analysis using a polyclonal antibody anti-RyR was also performed on spermatozoa (Figure 4). In CTR, the signal was weakly localized in the neck region (Figure 4A) and sometimes along the sperm tail; after incubation with F_4_, the label was clearly localized in the midpiece (Figure 4B) of the sperm; after incubation with F_4_ + D50, the signal newly appeared weak in the neck region of sperm (Figure 4C).

## 3. Discussion

Here, it was demonstrated that the presence of dantrolene, the chemical inhibitor of RyRs, reduced the effect of F_4_-NeuroPs on sperm motility. Thus, in spermatozoa, similarly to what was previously demonstrated in cardiomyocytes [18], the biological activity of F_4_-NeuroPs is supported, and it appears to involve RyR receptors.

This adds new information to the biological relevance of isoprostanoids [19]. The widely investigated F_2_-isoprostanes appear to be capable of inducing biological activity through the activation of receptors related to thromboxane A_2_ receptor [20,21]. In semen, our data support the relevance of F_4_-NeuroPs, not only as a biomarker of lipid oxidative damage but also as a lipid mediator involved in motility regulation and probably in other physiological processes. In spermatozoa F_4_-NeuroPs seem to act on RyRs [18]. The calcium homeostasis is regulated by two main Ca_2+_ channels: Orai1, which regulates store-operated calcium influx [22], and CatSper [23,24] which controls the intracellular calcium concentration; however, RyRs are present and involved in calcium transport [25].

In this study, the involvement of F_4_-NeuroPs on RyR stimulation was evaluated using dantrolene as a referred chemical inhibitor of RyR1 [26]. In our experimental protocol, the minimum effective dose of dantrolene in affecting sperm motility was established and applied to evaluate the inhibitory effect on the biological activity of F_4_-NeuroPs. The involvement of RyRs in regulating sperm motility was hypothesized by the inhibitory effect of dantrolene that, at a concentration of 50 μM, causes a derangement of normal sperm motility. This dysregulation of motility is manifested by an increase in in situ motility and the appearance of circular motility and by an increase in immotility. These types of motilities represent alterations of physiological motility, therefore resulting in non-functional, and might be due to altered polarization of the sperm membrane and/or alteration in calcium influx [27,28]. Other inhibitory molecules, for example genistein, may modulate sperm motility and acrosome reaction [29].

The concomitant presence of F_4_-NeuroPs and dantrolene indicates that the previously demonstrated effect of increasing rapid progressive motility in spermatozoa treated with F_4_-NeuroPs is not only influenced but also replaced by the opposite effect of motility reduction. This leads to the hypothesis that the two molecules compete for binding to the same receptor or similar receptors that regulate spermatozoa movement. This conclusion is possible from the experimental setting in which D50 is used, which in fact has been defined here as the minimum effective dose. In the presence of D100, the rapid motility is so severely reduced as to not allow accurate conclusions.

Although previously demonstrated that F_4_-NeuroPs can induce sperm capacitation, the inhibitory effect on this process by the simultaneous presence of dantrolene cannot be evaluated here because the experimental observations were conducted in a time span that is not sufficient to activate capacitation. However, the PSA staining showed that the clear localization in intact acrosomes from controls was different after the incubation with F_4_-NeuroPs and F_4_-NeuroPs and D50 in which the label appeared faint, sometimes indicating altered acrosomes, suggesting a step probably evolving into an acrosomal reaction. The choice of observation times was evaluated and established with the awareness that the binding dynamics to cellular receptors by F_4_-NeuroPs and dantrolene can change quickly and repeatedly. Therefore, since the main objective of this study was to evaluate the involvement of RyRs in the biological action of F_4_-NeuroPs, the experiments were performed with a timing that favored the possibility of evaluating the concomitant effect of F_4_-NeuroPs and dantrolene. The strong signal of RyRs in the sperm midpiece after F_4_-NeuroP incubation compared with that found in controls and in samples with F_4_-NeuroPs and D50 samples may suggest the involvement of mitochondria in the increase in sperm motility due the effect of to this metabolite.

Here, the dose of F_4_-NeuroPs used is the same as that already selected through previous studies conducted to evaluate its effect on spermatozoa movement and capacitation [10,11]. The concentration of dantrolene was evaluated within ranges already tested [30] and here assessed and selected based on the modulation of sperm motility and vitality. Precisely, to refer to already-established data, the quantity of F_4_-NeuroPs used here is expressed in ng to make the connection with previous studies clear. However, considering the molecular weight, F_4_-NeuroPs were used in the order of 20 μM (precisely, 19 μM).

Several ion channels are implicated in the motility pathway control in mammalian spermatozoa, and their content and regulation mechanisms change in mammalian species [31]. In humans, calcium and potassium conductance are associated with fertility [32].

Our data may indicate that alterations in RyR function negatively affect human sperm physiology, even if sperm vitality was not influenced. Different ion channels relate to sperm motility and act in sequence [33,34]. The functional importance of ion channels such as CatSper in sperm motility was conceived as a tool to be investigated not only in the field of male infertility but also in contraception [35].

## 4. Materials and Methods

### 4.1. Donors

Semen samples of thirty-six normozoospermic (sperm parameters > 25th percentile of World Health Organization guidelines [17]) donors (23–36 years old) attending the Department of Molecular and Developmental Medicine, University of Siena (Italy), were recruited for this study. All the donors signed a written informed consent agreeing that their semen samples would be only used for scientific purposes. The protocol was approved by the Ethic Committee of Siena University Hospital, ID CEAVSE 25612.

### 4.2. Semen Analysis and Sperm Selection

Semen samples were collected by masturbation after 3–5 days of sexual abstinence and analyzed after liquefaction for 30 min at 37 °C. Semen analysis was carried out according to the WHO guidelines [17] evaluating sperm concentration and motility. Eosin Y assay was used to assess sperm vitality.

In order to recover the most homogeneous sperm population possible, the swim-up selection technique was used. Briefly, 1 mL of Sperm Washing Medium IrvineScientific^®^ (Santa Ana, CA, USA) was stratified on 1 mL of semen in sterile conical centrifuge tubes tilted at a 45° angle. After an incubation of 45 min at 37 °C, the uppermost part, rich in motile spermatozoa, was recovered and used for the experiments.

### 4.3. 4-F_4t_-NeuroPs and 10-F_4t_-NeuroPs

As 4- and 10-F_4t_-NeuroPs are not commercially available, they were synthesized in-house (Institut des Biomolécules Max Mousseron, Montpellier, France), as previously described [10].

### 4.4. Step 1: Effects of Different Dantrolene Concentrations on Selected Sperm Population

In each experiment, the upper fraction recovered from swim-up of 18 semen samples was divided into aliquots treated with 10 μM, 30 μM, 50 μM, and 100 μM dantrolene (Merck, Sigma-Aldrich, Cod. D9175, Merck KGaA, Darmstadt, Germany) D10, D30, D50, and D100, respectively, the chemical inhibitor of RyRs, to test its effects on sperm motility and vitality. For these treatments a stock solution of dantrolene 430 μM diluted in swim-up medium was prepared by sonication and heating.

One untreated aliquot was used as control. After 30 min of incubation at room temperature, sperm motility and vitality were assessed as previously reported.

### 4.5. Step 2: Effects of Combined Treatment of Dantrolene and F_4_-NeuroPs on Selected Sperm Population

In each experiment, the upper fraction recovered from swim-up of another 18 semen samples was divided into the following aliquots and incubated at room temperature for 30 min:

- Untreated aliquot used as control (CTR);
- Aliquot treated with 7 ng of F_4_-NeuroP solution made up by 4-F_4t_-NeuroP and 10-F_4t_-NeuroP 1:1 [12] (F_4_);
- Aliquot treated with a combination of 7 ng of F_4_-NeuroP solution and 50 μM of dantrolene (F_4_ + D50);
- Aliquot treated with a combination of 7 ng of F_4_-NeuroP solution and 100 μM of dantrolene (F_4_ + D100).

At the end of the incubation, sperm motility was classified as rapid motility, in situ motility (non-progressive), and circular motility (circular movement of sperm as around a point).

The aliquots were washed with phosphate-buffered saline (PBS) and centrifuged and the pellet was recovered. Then, spermatozoa were smeared on glass slides and fixed in methanol at −20 °C for 20 min and in acetone at −20 °C for 5 min; the slides were used for the evaluation of the acrosome and in the immunofluorescence experiment for the localization of RyRs in F_4_ + D50.

### 4.6. Evaluation of Acrosome Status with Pisum sativum Agglutinin

TRITC-conjugated *Pisum sativum* agglutinin (PSA, Vector Laboratories Inc., Burlingame, CA, USA), recognizing the carbohydrate part of glycoprotein, is used to highlight the acrosome status. Smeared fixed slides were rinsed in PBS for 10 min, incubated for 30 min (in the dark at room temperature) in TRITC-PSA solution diluted 1:1000 in PBS, and rinsed again in PBS for 15 min. Then, a solution of 4′,6-diamidin-2-fenilindolo (DAPI; Vysis, Downers Grove, IL, USA) diluted 1:20,000 in PBS was used to stain sperm nuclei, incubating slides for 10 min in the dark at room temperature. After rinsing in PBS, the slides were mounted with 1,4-diazabicyclo [2.2.2]octane (DABCO; Sigma-Aldrich, Milan, Italy) and observed with a Leica DMI 6000 fluorescence microscope (Leica Microsystems, Wetzlar, Germany). A Leica AF6500 Integrated System for Imaging and Analysis (Leica Microsystem, Wetzlar, Germany) was used for image acquisition. Spermatozoa showing homogeneously stained red caps were classified as acrosome-intact spermatozoa or as acrosome absent, when the signal was absent, or as altered acrosome, when the label appeared faint. This reduced label may be associated with a state of pre-acrosomal reaction.

Three samples were evaluated.

### 4.7. Immunolocalization of Ryanodine Receptor

Smeared fixed slides were rinsed in PBS for 10 min, treated with blocking solution [1% PBS–bovine serum albumin (BSA), 5% normal goat serum (NGS)] for 20 min at room temperature and then incubated overnight at 4 °C with a primary rabbit anti-RyR polyclonal antibody (Invitrogen, Thermo Fisher Scientific, Carlsbad, CA, USA) diluted 1:500 in 0.1% PBS-BSA and 1% NGS. The following day, the slides were washed 3 times in PBS with 0.1% Tween 20 and incubated for 1 h at room temperature with a secondary goat anti-rabbit antibody Alexa Fluor^®^ 488 conjugate (Invitrogen, Thermo Fisher Scientific, Carlsbad, CA, USA) diluted 1:500 in 0.1% PBS-BSA and 1% NGS. Nuclei were stained with DAPI (Vysis, Downers Grove, IL, USA) solution diluted 1:20,000 for 10 min at room temperature; then, the slides were washed in PBS and mounted with DABCO (Sigma-Aldrich, Milan, Italy). A negative control was made by omitting the primary antibody in order to reveal non-specific binding of the secondary antibody. The slides were observed under a Leica DMI 6000 fluorescence microscope (Leica Microsystems, Wetzlar, Germany) and images were acquired using the Leica AF 6500 Integrated System for Imaging and Analysis. For each sample, approximately 100 sperm were evaluated, and the presence of labeling was recorded.

Three samples were evaluated.

### 4.8. Statistical Analysis

The normal (Gaussian) distribution of the data was assessed by D’Agostino–Pearson or Shapiro–Wilk normality tests. In the non-normal distribution of data, the results were reported as medians and 95% confidence interval (C.I.) or interquartile ranges; multiple comparisons were performed by one-way analysis of variance (ANOVA), applying the Kruskal–Wallis test followed by the non-parametric Mann–Whitney test. The statistical significance was defined as *p* < 0.05. The data analysis was carried out by the Graph-Pad Prism 8.4.2 statistical software package.

## 5. Conclusions

In conclusion, although this is not intended to be a pharmacokinetic study, the use of the chemical inhibitor of RyRs has allowed the evaluation of the relevance of RyRs in the regulation of sperm movement and shown that the effect of F_4_-NeuroPs on sperm motility is linked to the stimulation of RyRs. Therefore, in sperm cells, F_4_-NeuroPs appear capable of exerting biological activity that involves RyRs.

## Figures and Tables

**Table 1:** Medians [lower and upper 95% C.I. values] of progressive rapid, in situ, and circular motility percentage in sperm incubated with 50 μM and 100 μM dantrolene (respectively, D50 and D100) and/or 7 ng F_4_-NeuroPs. Kruskal–Wallis test *p* ≤ 0.0001, *n* = 18. The Mann–Whitney test was applied for post hoc pairwise comparisons. Statistical significance for pairwise comparisons is shown in the *p* value columns.

	CTR	F_4_	F_4_ + D50	F_4_ + D100	Statistics
Rapid progressive motility (%)	55.00 [54.06–55.94]	65.00 [63.23–66.10]	38.00 [36.25–38.42]	8.00 [7.79–8.87]	F_4_ vs. CTR; F_4_ + D50 vs. F_4_; F_4_ + D50 vs. CTR; F_4_ + D100 vs. F_4_; F_4_ + D100 vs. CTR; F_4_ + D50 vs. F_4_ + D100; *p* < 0.005
In situ motility (%)	1.00 [0.79–1.87]	2.00 [1.12–2.21]	6.00 [5.79–6.87]	7.00 [6.79–7.87]	F_4_ + D50 vs. F_4_; F_4_ + D50 vs. CTR; F_4_ + D100 vs. F_4_; F_4_ + D100 vs. CTR; F_4_ + D50 vs. F_4_ + D100; *p* < 0.005
Circular motility (%)	0.00 [0.00–0.00]	0.00 [0.00–1.00]	4.00 [4.00–4.00]	5.00 [4.12–5.21]	F_4_ + D50 vs. F_4_; F_4_ + D50 vs. CTR; F_4_ + D100 vs. F_4_; F_4_ + D100 vs. CTR; *p* < 0.005 F_4_ + D50 vs. F_4_ + D100; *p* < 0.05

## Data Availability

The data generated and analyzed during this study are included in this published article and are available from the corresponding author.

## Abbreviations

The following abbreviations are used in this manuscript:

F_4_-NeuroPs	F_4_-Neuroprostanes
DHA	Docosahexaenoic acid
RyRs	Ryanodine receptors
PSA	*Pisum sativum* agglutinin
PUFAs	Polyunsaturated fatty acids
CatSper	Cation channel of spermatozoa
WHO	World Health Organization
PBS	Phosphate-buffered saline
BSA	Bovine serum albumin
NGS	Normal goat serum
DAPI	4,6-Diamidino-2-phenylindole
